# Individual Spawning Duration of Captive Atlantic Bluefin Tuna (*Thunnus thynnus*) Revealed by Mitochondrial DNA Analysis of Eggs

**DOI:** 10.1371/journal.pone.0136733

**Published:** 2015-08-28

**Authors:** Ana Gordoa, Nuria Sanz, Jordi Viñas

**Affiliations:** 1 Department of Marine Ecology, Centro de Estudios Avanzados de Blanes, Spanish National Research Council (CSIC), Blanes, Girona, Spain; 2 Laboratori d'Ictiologia Genètica, Departament de Biologia, Facultat de Ciències, Universitat de Girona, Girona, Spain; The Ohio State University, UNITED STATES

## Abstract

This study presents the first results on Atlantic bluefin tuna (*Thunnus thynnus*) individual spawning duration and its short-term temporal behavior. The study was based on direct measurements resulting from mtDNA analysis of the offspring of spawners held in transport cages during the 2013 spawning monitoring survey in Balearic waters. The number of females consisted of approximately 259 individuals with an average weight of 186 kg. The survey began on May 22 and ended on July 3. Spawning started on May 30 and was observed every night afterwards. The sampling of eggs for genetic monitoring was conducted for 9 days interspersed from the beginning of spawning to the end of the survey. A total of 946 eggs were analyzed and revealed 129 different haplotypes; 77 of these were not previously detected in the Mediterranean. A total of 69 haplotypes were observed in more than one spawning event and those with higher frequency lasted their maximum possible duration. The haplotypes present at the beginning of spawning were also identified at the end of the sampling, indicating a minimum spawning duration of 34 days, and individual annual fecundity was estimated at around 1290 eggs gr^-1^. These results differed from those generally presumed until now and are indicative of a much higher fecundity. Females exhibited a regular spawning schedule but with the capacity to shift the spawning hour during the spawning season. These results were observed for the eastern population of Atlantic bluefin tuna and before extrapolating to the western population, their validity should be proved.

## Introduction

The Atlantic Bluefin tuna (*Thunnus thynnus*) is a highly migratory species, widely distributed throughout the North Atlantic Ocean and the Mediterranean Sea. This species comprises two different populations [1]: western and eastern Atlantic, which are managed separately by the International Commission for the Conservation of Atlantic Tunas (ICCAT). These populations differ greatly in size, the eastern is considered to be around 10 times the size of the western [1,2]. The observed difference in population size may be increasing, particularly considering the latest ICCAT stock assessments [3] where a clear recovery was only observed for the eastern stock. The stocks share a wide range of feeding grounds [4], implying a wide spatial mixing that makes management more difficult. In addition, the results from recent studies appear to indicate that stock mixing might be higher than previously presumed [4,5], contributing to increasing the observed uncertainty in the western stock assessment [3] or hindering its recovery [6]. In spite of their spatial mixing, these populations are genetically distinct [7] as a result of separate reproductive basins, Mediterranean (hereinafter, Med) and Gulf of Mexico (hereinafter, GOM) for the eastern and western stocks respectively [4,8].

The reproduction of Atlantic Bluefin Tuna (ABFT, here after) is characterized by serial batch spawning and asynchronous oocyte development, distinctive of tunas [9]. The length of the reproductive period of ABFT populations differs from other tunas by its shortness [9]. The reproductive period varies between and within reproductive regions; from April to June in GOM [4,10,11], and within the Mediterranean basin it varies in correlation with a progressive east-to-west increase of the sea surface temperature throughout spring [12]. In the Western Mediterranean ABFT spawning has been reported from June to July [13–19] and a month in advance in the Levantine Sea [20].

The first studies on the reproductive biology of ABFT, traditionally approached by histological analysis, were published eight decades ago [21], while the first estimation of ABFT relative fecundity [17] was made four decades ago. The study of fecundity remains a key issue, as it is an essential reproductive feature for stock assessment, allowing a better estimation of the stock reproductive potential and of the intrinsic rate of population increase [22]. Although fecundity is related to recruitment variation [23], recruitment is the net output of egg production (fecundity) and offspring survival. Stock recruitment models are still widely used today despite their limited or null explanatory capacity, and in the particular case of ABFT these models have been proved highly unsatisfactory for both stocks [24]. However, they are still in use, but due to their inefficiency different recruitment scenarios had to be adopted for both stocks and are still used [3]. In 2009, and in order to provide scientific advice on the condition of ABFT, with respect to the criteria applied for commercial exploited aquatic species under CITES, an alternative method of estimating ABFT productivity was developed [25]. This alternative, called the potential rate of population increases, was first proposed by Jennings et al., [26] and expressed by fecundity at the length at which 50% of the stock attains maturity (L50) and its corresponding age (A50). The differences between ABFT stocks, in age at maturity and fecundity, resulted in differences of their potential rate of population increase, which was found to be appreciably higher for the eastern stock. However, fecundity comprised different specific terms, and when used indistinctly might lead to flawed conclusions. The fecundity at L50 considered for ABFT stocks [25] could be either the batch fecundity—total number of eggs spawned per batch [27]— or total fecundity—number of advanced oocytes at any time in the ovary [28]—but neither of these are indicative of annual fecundity. Thus, the apparent differences found between western and eastern stocks in their potential rates of increase should be questioned until annual fecundities are estimated for each stock.

The annual fecundity is defined as the number of eggs released per female and year [28] and should be estimated from the product of three parameters: the number of oocytes released per spawning event (batch fecundity), the spawning frequency and the individual duration of the spawning. The first two parameters for ABFT eastern stock are known and were estimated from histological analysis; batch fecundity around 59 eggs g^-1^ or 48 eggs g^-1^ in the most recent estimations, and with spawning periodicity of 1.2 days [29,30]. The above-mentioned spawning periods of ABFT, known for different reproductive areas, are inferred from the presence of spawners and larvae and from the results of histological analysis of gonad development. However, a key open issue is to ascertain the individual duration of spawning, the remaining unknown parameter for estimating the annual fecundity. In recent years, major advances have been made in tuna reproductive biology and also at an individual level, as a consequence of the introduction of studies on cultured tuna and new technologies, such as electronic tagging and genetics.

The genetic monitoring of captive spawning populations of tuna has been shown to be possible by analyzing the mitochondrial DNA (mtDNA) markers. Since the mtDNA control region of the genus *Thunnus* is highly polymorphic [31,32], it displays enough variation to discriminate among individuals [33]. These techniques have been successfully carried out in the broodstock of different species of cultured tuna such as Pacific Bluefin tuna [33–36] or Yellowfin tuna [37], providing information about their spawning frequency and periodicity at individual level, key issues for identifying the more fertile females for culture. However, as ABFT spawning was observed and defined quite recently [38], its culture is still in its early stages [39] and the genetic tracking has not yet been studied for either offspring or broodstock. Thus, the limited knowledge of ABFT individual spawning behavior comes from wild fish spawners tracked by remote sensing and inferred from the duration of a particular swimming behavior; first observed in GOM [11] and recently in the Mediterranean [40]. This inference is justified because the particular swimming behavior coincides with the spawning hours observed for the eastern population [41].

The direct observation of ABFT spawning in the natural system is a difficult task, and its long-term monitoring was unfeasible until tuna transport cages were revealed to be effective spawning observatories [38]. Their monitoring during four consecutive seasons provided relevant information on spawning behavior [41] but the individual spawning temporal behavior is still unresolved: neither the individual spawning extension nor the individual fidelity to spawning hours. The purpose of this investigation was to answer these questions by analyzing the variability of the mtDNA CR sequence of eggs spawned by ABFT adults held and monitored in a transport cage during the 2013 spawning season.

## Materials and Methods

**Fig 1:**
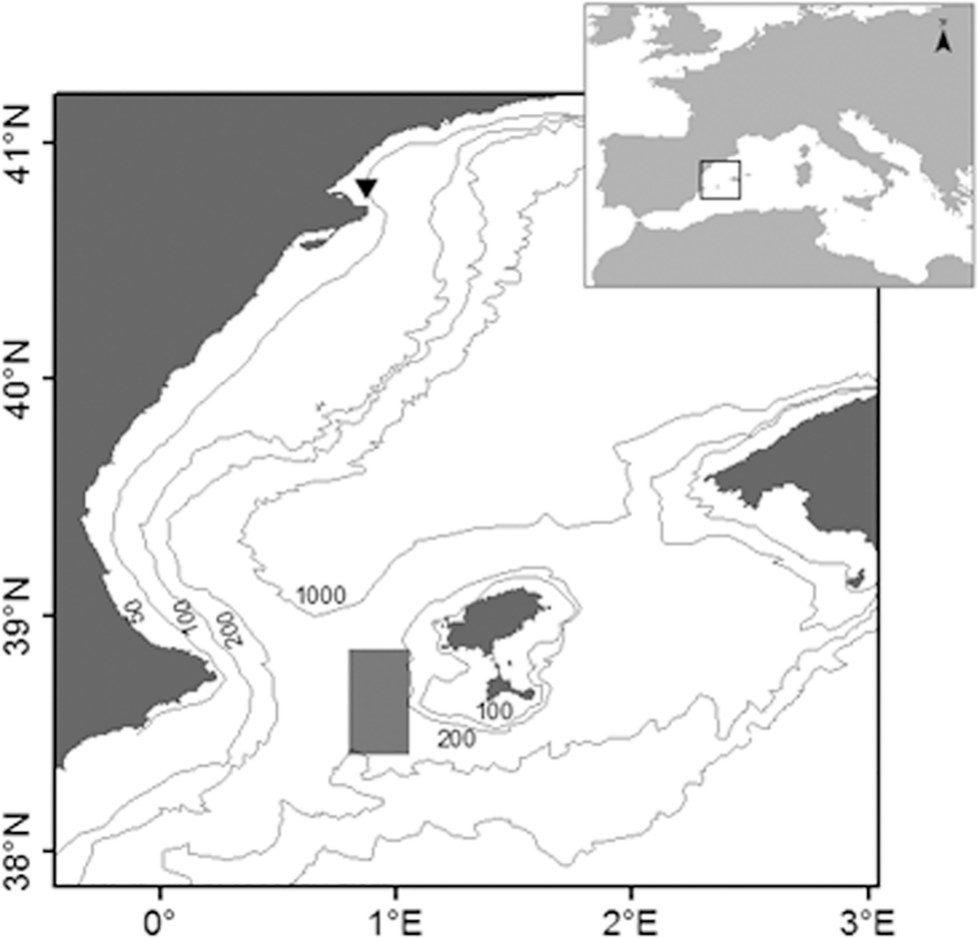
Map showing the sampling and farm locations. Primary location of the tuna transport cage during the spawning monitoring (█) and the location of the fattening facilities from where the captive tuna was transported every spawning season (▲).

Each spawning season, since 2009, a group of ABFT spawners captive in the Balfegó fattening facilities is transferred to a transport cage which is towed and transported to the Balearic ABFT spawning region for research purposes (Fig 1). The transport of tuna groups under monitoring required previous authorization from the Spanish Directorate-General for Fisheries. The study was carried out offshore, where no specific permission is further required. The cage is monitored from the middle of May to the beginning of July, covering most of the spawning season in the western Mediterranean [41], when it is transported back to the farm. The objective of this particular study, the mitochondrial DNA analysis of eggs, was included in the 2013 survey.

In 2013 the number of tuna transported was 563, with an average weight of 186 kg. The survey began on May 22^nd^ and ended on July 3^rd^. Egg sampling followed the plankton protocol for transport cages already tested successfully in earlier studies [38,41]. Samples were collected using bongo nets fitted with 0.3 mm-mesh nets deployed behind the transport cages at a depth of three metres. The towing speed of the transport vessels was constant at around 0.6 knots. Sampling time (local time = UTC + 2 hours) was established within the spawning time interval found for this species [41]. Three consecutive hours were sampled per night: 2:00–2:55 a.m., 3:00–3:55 a.m. and 4:00–4:55. Upon retrieval of the gear, plankton samples were immediately preserved in 5% buffered formalin. From each sample with eggs, a subsample of 2 ml of eggs was separately preserved in absolute ethanol. Semi-quantitative measurements of the egg volumes collected at each sampling station were estimated. The volume of eggs (in millilitres) collected at each station was estimated after settling in 250-ml translucent jars. At several stations 250-ml jars were too small to hold all the eggs collected, so the spare volume of eggs was measured before being returned to the sea or kept for hatching experiments.

**Fig 2:**
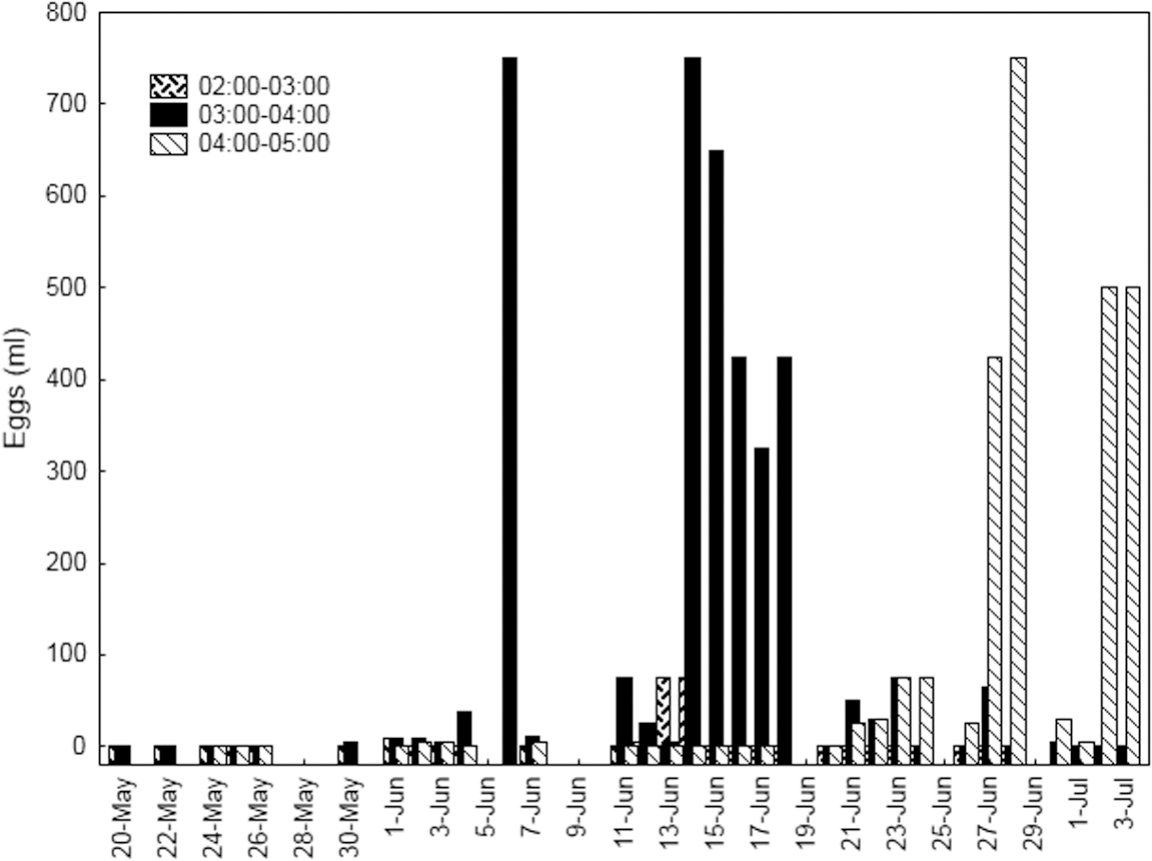
Temporal spawning pattern of ABFT monitored group. Volume (ml) of eggs collected per day and time interval from Atlantic bluefin tuna caged group in 2013 spawning survey.

The mitochondrial analysis of each spawning event was unaffordable and only eggs from a determined subset of samples were analysed. The selection criteria of the samples were grounded to reach the objectives set and based on the results of the daily spawning pattern (Fig 2). To achieve the first objective, the individual spawning duration, the first and the last observed spawning events were selected and in between samples every 4/7 days were also analysed. To determine if the spawning hour at the individual level was variable, three pair samples corresponding to spawning events at different hours in the same day were selected. A total of 12 spawning events corresponding to 9 different days were analysed. The minimum number of eggs analysed for each spawning event was 50 and doubled to 100 for events with higher spawning intensity. The eggs were preserved in 96% absolute ethanol until analyzed.

To avoid cross-contamination between eggs in the same sampling tube, previous to DNA extraction, all eggs were individualized and rinsed twice with 96% alcohol using 0.1 mm mesh. The cleansed eggs were DNA extracted individually using DNA E.Z.N.A.Mollusc DNA Kit (OMEGA Biotek, USA). Approximately 400 bp of the mtDNA control region was amplified and subsequently sequenced as described in Viñas and Tudela [32]. The sequences obtained were aligned, edited and checked for quality using the Geneious version 7.1.5 software (http://www.geneious.com, [42]). Haplotype inference was carried out using DNAsp version 5.10 [43] and haplotype diversity (*h*) was estimated in Arlequin version 3.5 [44]. Sequences were submitted to Genbank with accession numbers KR337331-KR337459.

## Results

A total of 946 eggs were analyzed. Of these, nine presented high sequence similarity with *Thunnus alalunga* and *Thunnus orientalis*, a consequence of the mtDNA introgression between *Thunnus* species as has been reported previously [45], and so these sequences were removed from the analysis. Then a total of 937 eggs were analyzed and revealed 129 haplotypes, and 53 of them were represented by one single egg. Haplotype diversity per sample (spawning event) ranged from 0.697 to 0.971 with average haplotype diversity for all samples of 0.965 (Table 1). It is important to point out the high percentage of haplotypes (60%) that have not previously been detected in the Mediterranean, a total of 77, with some of them observed in more than 3 spawning events. As expected, some of the most common haplotypes in the Mediterranean [45] were also present in our results (Table 2).

**Table 1:** Sampling and haplotipic details. Sampling dates and hours, number of analyzed eggs, number of haplotypes per sample, number of unique haplotypes (present in one single egg) and haplotypic diversity.

Date	Local time	Analyzed eggs	N° haplotypes	N° unique Haplotypes	Haplotypic diversity(SD)
**30 May**	3:00–4:00	66	11	5	0.798 (0.027)
**1 Jun**	2:00–3:00	60	8	4	0.697 (0.039)
**1 Jun**	3:00–4:00	66	6	0	0.801 (0.023)
**6 Jun**	3:00–4:00	109	39	18	0.946 (0.006)
**14 Jun**	3:00–4:00	107	30	16	0.933 (0.011)
**18 Jun**	3:00–4:00	113	41	17	0.967 (0.006)
**23 Jun**	3:00–4:00	58	18	8	0.909 (0.019)
**23 Jun**	4:00–5:00	49	14	5	0.901 (0.018)
**27 Jun**	3:00–4:00	51	20	10	0.922 (0.019)
**27 Jun**	4:00–5:00	109	31	16	0.939 (0.009)
**30 Jun**	4:00–5:00	56	23	13	0.930 (0.107)
**3 Jul**	4:00–5:00	93	42	21	0.971 (0.006)

**Table 2:** Time tracking of haplotypes with high frequency (> 3 spawning events).

	30 May 3–4	1 Jun 2–3	1 Jun 3–4	6 Jun 3–4	14 Jun 3–4	18 Jun 3–4	23 Jun 3–4	23 Jun 4–5	27 Jun 3–4	27 Jun 4–5	30 Jun 4–5	3 Jul 4–5
**Hap_9** ^b^	x		x	x	x	x	x		x	x	x	x
**Hap_1**	x	x		x	x	x	x	x		x		x
**Hap_32** ^b^	x			x	x	x	x		x	x	x	x
**Hap_4**		x		x	x	x		x	x	x	x	x
**Hap_31** ^b^				x	x	x	x		x	x	x	x
**Hap_3** ^b^		x		x				x	x	x		x
**Hap_50** ^b^				x	x	x	x	x				x
**Hap_23** ^a^				x		x	x				x	x
**Hap_27**					x	x				x	x	x
**Hap_47** ^a^				x	x	x				x		x
**Hap_58**				x	x	x	x			x		
**Hap_6**	x	x		x		x				x		
**Hap_7**		x		x		x		x		x		
**Hap_12**			x		x					x	x	
**Hap_14**			x			x	x				x	
**Hap_17**						x	x				x	x
**Hap_2** ^a^		x							x	x		x
**Hap_39**					x	x					x	x
**Hap_57** ^a^				x		x				x	x	
**Hap_90**	x					x				x	x	

^a^haplotypes not detected in the Mediterranean population.

^b^haplotypes most frequent (>3%) in the Mediterranean

**Fig 3:**
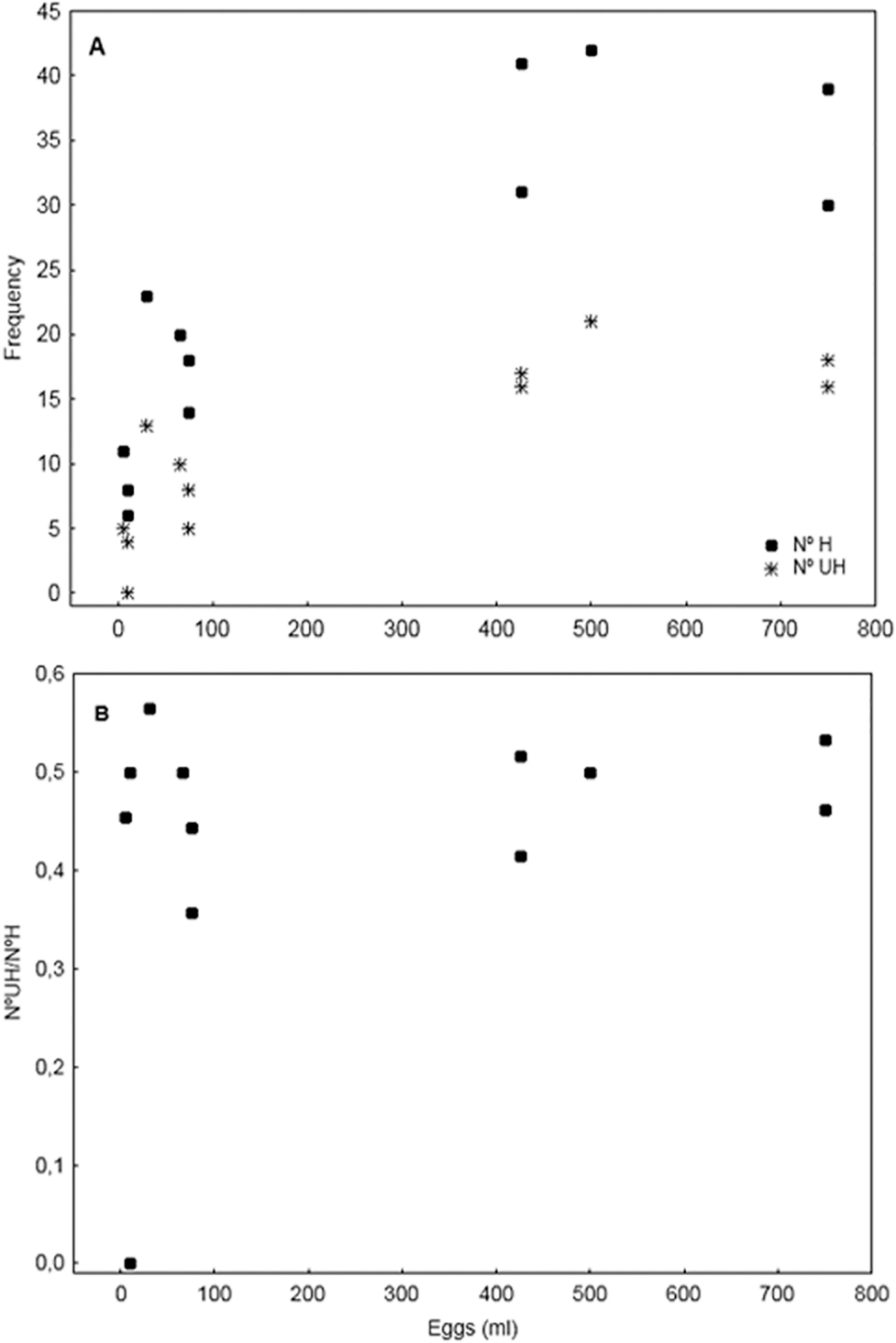
Haplotypes descriptors. Total haplotypes (N° H) and unique haplotypes (N° UH) as a function of spawning intensity (A) Frequency. (B) Proportion of.

The number of total haplotypes per spawning event, indicative of the number of females sampled in each spawning event, increased with the spawning intensity (Fig 3A) and a similar pattern was observed with the number of unique haplotypes per sample (one single egg per sample). Consequently, the relative frequency of unique haplotypes, which varies from 35% to 56% of the total, was independent of the spawning intensity (Fig 3B), excluding one single event with total absence of unique haplotypes. Those frequencies are indicative of the representativeness of each sample to identify the active number of females at each spawning event: the higher the percentage of unique haplotypes, the higher the sample’s representativeness.

**Fig 4:**
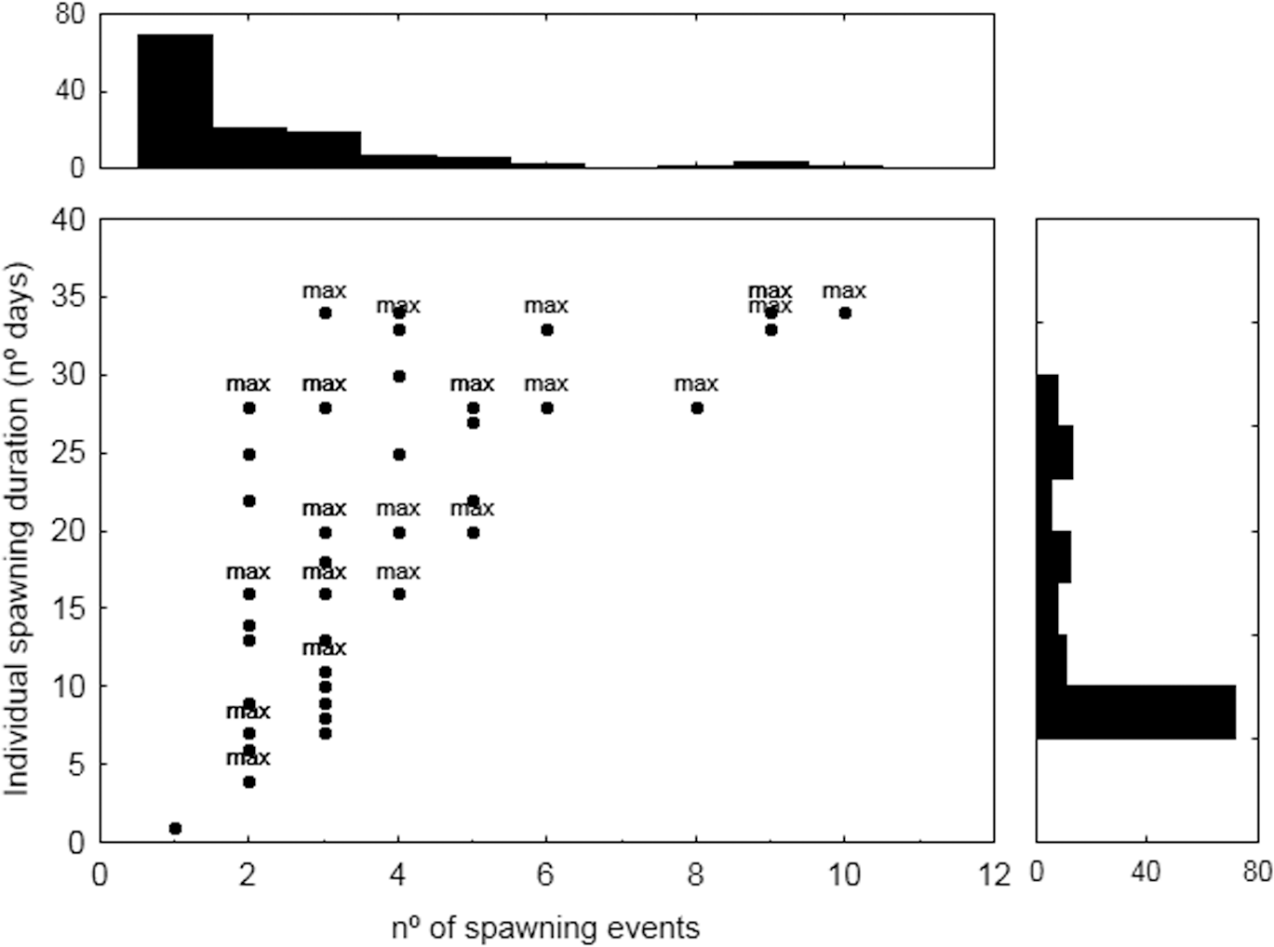
Duration of haplotypes as a function their spawning event frequency; at the sides the histograms of observed frequencies of spawning duration and spawning frequency.

The extent of the time interval at which each haplotype was observed could be considered as a proxy of the spawning duration of each female if sampling is under optimal conditions. It should be noted that the results of our estimations are highly limited by the length of the sampled period, which constrains the maximum duration to 34 days. Additionally, the small sample size rules out the possibility of sampling the offspring of all female spawners, and there is no likelihood of detecting all active females in each spawning event. The results showed that as the frequency increased (number of events each haplotype was identified) the minimum values of duration also increased, while the maximum duration was independent of the frequency (Fig 4). The maximum possible duration, the 34 days of survey duration, was observed in several haplotypes with sampling presence varying from 3 to 10. Individual haplotype duration is also limited by the date each haplotype was first identified in our sampling: the later it was identified, the shorter the estimations. However, considering the date of the first occurrence, more than fifty percent of the haplotypes extended up to the last sampling day, so they exhibit the potential maximum duration (Fig 4).

The annual individual fecundity was estimated on 1290 eggs gr^-1^ resulting from the product of batch fecundity and the number of batches during the individual spawning duration. The number of batches were estimated by dividing the estimated individual spawning duration (34 days) by the spawning interval, 1.2 days [29] and then multiplied by batch fecundity, 45,56 eggs gr^-1^ [46].

The results on the spawning hours shown in Fig 2 revealed that the spawning interval was quite narrow and did not vary randomly between days. The spawning was tuned or synchronized between 3:00–4:00 during the first 3 weeks of June, but minor spawning pulses were observed from 2:00 to 3:00 during three days. Throughout the third week of June the spawning intensity decreased and it took place during two consecutive hours, from 3:00 to 4:00 and from 4:00 to 5:00. The results over the last week showed an important upturn in spawning and the spawning hour was highly synchronized and restricted from 4:00 to 5:00. At the individual level, the results of mtDNA of eggs spawned at different hours showed the same pattern (Table 2). At the beginning of spawning, the first two days, the same females spawned indistinctly at 3:00–4:00 or at 2:00–3:00, then converging rapidly to the second hour until the third week. In the third week the females shifted their spawning towards the third spawning hour, 4:00–5:00. It is notable that the day before they definitively shifted to the later hour some females had two spawning pulses at hours, the old and the new one.

## Discussion

This study presents the first results on ABFT individual spawning duration and temporal behavior, based on direct measurements resulting from mtDNA analysis of the offspring of spawners held in transport cages. This study reveals once again the capability of tuna transport cages as monitoring observatories and experimental platforms [38,41]. The results are discussed and contrasted with those previously inferred from indirect approaches [11,40]. However, the discussion of these results requires a preliminary overview concerning their representativeness.

The size of the captive tuna was substantially higher than in studies of a similar nature [33,36,37] and therefore also the potential number of released eggs. The number of captive females was around 259, 46% of the captive group, according to the sex ratio reported for this region, period and size of tuna [29,47]. In the Balearic waters, 84% of the female spawners’ population contributes to the daily spawning [29], which in this study represent 217 females. The potential numbers of eggs released daily per female result from the product of its individual weight and the batch fecundity. The average weight of captive tuna was around 186 kg and the batch fecundity for the ABFT eastern population is 45.56 eggs gr^-1^, in accordance with the latest and most conservative figure [46]. Consequently, the daily egg production of each captive female will be around 8.5 million eggs and the potential daily egg production of the whole captive group close to 2000 million. Thus, the fraction taken for genetic analysis represented an infinitesimal fraction of the total number of eggs potentially produced at each spawning event. These figures are essential to frame the inference of our results and are also essential in any study of a similar nature. On the other hand, the high haplotype diversity of ABFT in the Mediterranean (above *h* = 0.992, [45,48]) and the low probability (p = 0.008) of sampling in the wild population two individuals with the same haplotype [45,48] simplify the interpretation of the results. Therefore, eggs with the same haplotype could be considered spawned by the same female parent. This was also supported by the low levels of haplotype diversity in our samples, result of sampling siblings and consequently significantly lower (*p* = 0.009) than the average haplotype diversity found in the Mediterranean (above 0.991; [45,48,49]). In addition, there was a notably high number of new haplotypes found in this study, indicating that we are still in the early stages of analysis of ABFT haplotypes.

A total of 129 different haplotypes were identified in this study and they are indicative of the minimum number of active spawning females, c.a. 50% of the total captive females. Thus, despite the limitations of the sample size, the proportion of identified females was substantial, suggesting that most females could be active spawners. Furthermore, the proportion of females identified from their offspring was similar to those reported for other tuna species in similar studies [33,37]. However, at each spawning event the representativeness of the analyzed sample was low; the maximum number of haplotypes identified in one spawning event (42) scarcely represented the 16% of the total captive females, but this flaw did not prevent achieving the purposes outlined in this study. The specific goal line was to probe deeper into the individual reproductive parameters and temporal behavior, and particularly into the determination of individual spawning duration and spawning hour. These specific objectives required collecting eggs from the same females in different spawning events and this was achieved. The proportion of females identified in more than one spawning event was 53%, and 20 females could be identified in more than 3 events. The results showed that all the females with high frequency of occurrence (>3 spawning events) displayed similar temporal patterns, which made it possible to attain the predefined objectives.

The results showed that most females displayed their maximum feasible duration: generally they were present until the end of the sampling, but the individual durations were constrained by the first date of appearance of each haplotype. It is also worth mentioning that out of the total of the 20 females detected at the beginning of the spawning, May 30th–June 1st, 35% were also detected on the very last day of sampling (July 3rd). These results indicate that the individual spawning duration can last over a minimum of 34 days. This estimation is limited by the end of the sampling, before the end of spawning, so the individual spawning duration might be longer, but also variable, because the lengthening of individual spawning can vary with age, longer as they get older, as observed in different species of multiple-batch spawners [50–53]. Thus, we cannot rule out this possibility in ABFT, especially considering that larger tuna seem to arrive and to leave the spawning grounds earlier and according to length frequency from Spanish traps [54]. Nonetheless, the results clearly showed that the individual spawning period is longer than the ones might be inferred from the swimming behavior of wild spawners. Differences in the reproductive potential between wild and cultured tuna may exist despite the similarity observed in their daily spawning behavior [41].

Electronic tags have been proved essential for studying ABFT spatial distribution and behavior as a particular nighttime diving behavior, which was reported solely during the breeding season and in the spawning grounds, and consequently linked to spawning behavior. This particular behavior was first observed in the western population during the spawning season in the GOM: the duration was first estimated in two weeks [55] and later around 17 ± 7d [11]. Recently, in 2012, a similar behavior has been observed in the Balearic spawning ground, with an average duration of 23.9 ± 4.1 d [40] with maximum around 31 d, which is comparable to the spawning duration observed in this study. Nevertheless, it is likely that both figures are underestimating the real spawning duration because the electronic tags were released after the onset of spawning, at least 10 days after the beginning of spawning was observed in 2012 [41], and in the present study the sampling ended before the spawning ceased. The diving behavior was associated with ABFT spawning because it was only observed in the spawning grounds, and at least for the eastern stock additional studies corroborates the hypothesis: in the Balearic spawning grounds, where the spawning hour interval has been clearly defined [41] and comprises the hours on which the particular swimming was observed [40]. Thus, the western and eastern differences in diving duration can be actual differences in their reproductive parameters. Another difference between western and eastern diving behavior, according to the data published [11,40], is the amplitude of the spawning hour interval; up to ten hours in the GOM and a maximum of three hours in Balearic waters.

Although the studies on the reproductive behavior of the eastern population revealed that the diving behavior [40] and the spawning [41] match in the hour interval, it should be emphasized that they differ in duration. While, according to published information [40], diving lasted 90 min on average, the spawning events take place in less than 15 min [41]. It has been hypothesized that this diving may play an important thermoregulatory role during spawning and associated with courtship behavior [40]. This is consistent with the courtship swimming behavior observed in captive yellowfin tuna: the duration was similar (1 to 4 h) and was prior to actual spawning, which took place in less than 90 sec. [56]. Thus, as courtship swimming behavior it would take place before the spawning but as a thermoregulatory mechanisms would be more necessary after the spawning. More research is needed to determine the functionality of this swimming behavior as well as to explore if it is gender-related. In a recent comparative study of reproductive status between the two populations [46], the authors found differences in batch fecundity, 62% higher in the eastern (45.56 eggs. g^-1^) than in the western (28.14 eggs. g^-1^), but the difference could not prove statistically significant. Similarly, these authors did not find differences in the spawning frequencies between populations. The difference in the reproductive parameters of the two populations are continuously under debate, and in particular the age at maturity, an important parameter estimated around 3–4 y for the eastern [57,58] and 8–14 for the western [59,60]. However, in a recent study it has been suggested that the western population matures at a much younger age, similar to that one considered for the eastern [61].

The results on the spawning time of the captive group corroborate the hour interval (2:00–5:00) found in previous studies [38,41]. At the individual level each female had the potential to spawn at different hours but not haphazardly through the spawning period. Before the 23^rd^ June, spawning was synchronized from 3:00 to 4:00 and after the 27^th^ from 4:00 to 5:00, and some females could be tracked on both periods. Of high interest is the transition period, between the 23^rd^ and 27^th^ of June, when females could spawn at both hour intervals. This is indicative that each female had the capability to spawn in two separated pulses, and they apparently did so when they changed the spawning hour. The three-period spawning pattern was previously observed, but the possibility that the same females were contributing to the spawning for so many weeks was disregarded [41]. Furthermore, the shift in spawning hour was used to infer the individual spawning duration, c.a. 21 days, but this proved to be wrong according to the results presented in this study.

This study, the first genetic monitoring of ABFT offspring, has provided the first approximation of individual spawning duration for ABFT eastern population, the missing parameter for estimating the individual annual fecundity of this population, c.a. 1289 egg.g^-1^. There are no bases for extrapolating the results presented here to the western stock: in fact the differences in spawning-swimming duration found in other studies indicated the contrary. In addition to molecular techniques, further studies in electronic tagging will help to determine individual spawning duration for the western population and intra and inter variability of the two populations.
